# Assessment of Strategic Healthcare Purchasing Arrangements and Functions Towards Universal Coverage in Tanzania

**DOI:** 10.34172/ijhpm.2022.6234

**Published:** 2022-08-01

**Authors:** August Kuwawenaruwa, Suzan Makawia, Peter Binyaruka, Fatuma Manzi

**Affiliations:** Ifakara Health Institute, Dar es Salaam, Tanzania.

**Keywords:** Strategic Purchasing, Contracting, Insurance, Tanzania

## Abstract

**Background:** Strategic health purchasing in low- and middle-income countries has received substantial attention as countries aim to achieve universal health coverage (UHC), by ensuring equitable access to quality health services without the risk of financial hardship. There is little evidence published from Tanzania on purchasing arrangements and what is required for strategic purchasing. This study analyses three purchasing arrangements in Tanzania and gives recommendations to strengthen strategic purchasing in Tanzania.

**Methods:** We used the multi-case qualitative study drawing on the National Health Insurance Fund (NHIF), Social Health Insurance Benefit (SHIB), and improved Community Health Fund (iCHF) to explore the three purchasing arrangements with a purchaser-provider split. Data were drawn from document reviews and results were validated with nine key informant (KI) interviews with a range of actors involved in strategic purchasing. A deductive and inductive approach was used to develop the themes and framework analysis to summarize the data.

**Results:** The findings show that benefit selection for all three schemes was based on the standard treatment guidelines issued by the Ministry of Health. Selection-contracting of the private healthcare providers are based on the location of the provider, the range of services available as stipulated in the scheme guideline, and the willingness of the provider to be contracted. NHF uses fee-for-service to reimburse providers. While SHIB and iCHF use capitation. NHIF has an electronic system to monitor registration, verification, claims processing, and referrals. While SHIB monitoring is done through routine supportive supervision and for the iCHF provider performance is monitored through utilization rates.

**Conclusion:** Enforcing compliance with the contractual agreement between providers-purchasers is crucial for the provision of quality services in an efficient manner. Investment in a routine monitoring system, such as the use of the district health information system which allows effective tracking of healthcare service delivery, and broader population healthcare outcomes.

## Background

**Key Messages**

** Implications for policy makers**

- Investment in a routine monitoring system, such as the use of the district health information system which allows effective tracking of healthcare service delivery, and broader population healthcare outcomes.
- The government intention towards Single National Health Insurance (SNHI) not only calls for harmonization of the health insurance schemes across the country but also the healthcare purchasing functions including resources collection and pooling; healthcare benefits provided; payment mechanisms and data management systems.
- Communication of healthcare strategic purchasing arrangements and functions is crucial for the understanding of the interactions and accountability of the purchasing functions to relevant stakeholders, including the government, insurance schemes, and healthcare providers as well as the citizens.

** Implications for the public**

Enforcing compliance with the contractual agreement between providers-purchasers is crucial for the provision of quality services to the citizens in an efficient manner. Nonetheless, it is crucial to ensure efficient use of limited healthcare resources in meeting the needs of the population without jeopardising service provision and sustainability of the financing schemes. Direct resources to priority areas of health service delivery to ensure equitable access to minimum essential healthcare benefits package to the population and financial protection. Strengthen coordination between the entities managing the purchasing functions is important in improving access and utilization of quality healthcare as well as in ensuring equity. Healthcare financing reforms are taking place in the country, hence, purchasing functions should be reviewed to increase the potential in accelerating the country’s progress towards universal health coverage (UHC).

Strategic health purchasing in low- and middle-income countries has received substantial attention as countries aim to achieve universal health coverage (UHC), by ensuring equitable access to quality health services without the risk of financial hardship.^1,2^ An increase in public funds for health and expansion of social protection has been recognized as a means of achieving financial protection.^3^ Initially, efforts were directed towards resource generation while minimal efforts were directed on how best the pooled resources could be transferred/allocated to service providers.^4^ There have been challenges in clearly defining services covered, specifying service provision guidelines, provider payment rates, reimbursement mechanisms, and accountability of the limited resources generated.^4,5^

 A transparent contractual agreement between purchasers and providers is important for accountability in the provision of healthcare services. Contractual agreements form the basis for establishing standards, provider performance targets, and monitoring service provision competence and encourage providers, to comply with the agreements, such as ensuring sufficient numbers of skilled staff are available to meet population healthcare needs.^6^ It is important to monitor and enforce the contractual agreements with the healthcare providers to ensure the desired goal for the agreements is met.^7^ Contracting refers to the process that specifies what is purchased from healthcare providers and has been found to improve access to health services for the citizens.^8^ Healthcare purchasing is considered strategic if uses incentives to limit the provision of services which are expensive to provide, guarantees a minimum volume of services to be purchased; alternatively, a maximum volume may be set in order to limit total cost, pays relatively low prices for high-cost but low-priority services and links some part of payment to performance.^5^

 There is little evidence published from Tanzania on healthcare purchasing arrangements and functions. This study analyses purchasing functions and what is required for strategic purchasing among the three healthcare insurance schemes in Tanzania – National Health Insurance Fund (NHIF), Social Health Insurance Benefit (SHIB), and improved Community Health Funds (iCHF). Understanding such purchasing functions and what is required for strategic purchasing provides evidence to inform policy decisions to ensure limited financial resources generated and pooled at various levels are used in a way that optimally enhances the achievement of UHC.

###  Description of Key Features of Tanzania’s Health System

 In Tanzania, the health system has a mix of public, private for-profit, and private not-for-profit (faith-based) healthcare providers. Service provision is dominated by the public sector with facilities organized in a pyramidal structure, dispensaries being the lowest level of service delivery, followed by the health centres and district hospitals.^8,9^ In 2015, there were 6640 dispensaries of which 4554 (69%) were government owned, out of 701 health center 518 (74%) were government owned and out of 108 district hospital 70 (65%) were government hospitals in Tanzania.^10^ In districts which have no public hospitals, the government has entered into a service agreement with hospitals owned by faith-based organization to serve as district designated hospitals under the umbrella of public-private partnerships.^8,11^ Above the district hospitals, there are regional referral hospitals, zone hospitals, specialized hospitals, and national hospitals.^8^

 Health financing in Tanzania, is highly dependent on Development Partners, and there is still a gap in financial resource. Public health spending account for 34%, development partners 36%, health insurance schemes 8%, and out-of-pocket payment 22%.^12^ Out-of-pocket payment is still high compared to health insurance financing contribution to the total health expenditure in 2019/2020,^13^ imposing a financial risk to households, in particular the most vulnerable. Previous studies have shown that, Tanzania has the highest share of households spending in excess of 25% of their non-food expenditures on healthcare. In terms of intensity of catastrophic payments, the mean positive overshoot is remarkably high at the 25% threshold of the non-food budgets.^14,15^ In 2018, it was estimated that about 14 million citizens lived below the national poverty line of TZS 49 320 per adult equivalent per month and about 26 million (about 49% of the population) lived below the $1.90 per person per day international poverty line.^16^ A household survey conducted in the country to assess the correlates of out-of-pocket and catastrophic health expenditures found that the mean out-of-pocket payment health expenditures among the adult participants was US $2.2 (standard deviation 9.5).^14^ These costs have implication on the ability of the households to access healthcare services. General tax revenue and support from development partners are pooled at the central level and allocated to various sectors.^17^ For the health sector, these resources are pooled as the health sector basket funding for financing services aimed at the improvement of population health outcomes. Evidence suggests inequity in access and utilization of healthcare services as the poorest segment of the population access a lower proportion of services relative to their need for healthcare.^18^

 Tanzania has multiple fragmented health insurance schemes targeting different segments of the population with different characteristics.^19,20^ NHIF initially designed for public sector workers has recently opened up to informal sector enrolment. NHIF citizens willing and able to pay the premium can join NHIF. Funds are pooled centrally and managed by the NHIF board to purchase healthcare services from various healthcare providers.^21^ SHIB is managed by the National Social Security Fund (NSSF) as one of seven benefits offered to its members who are mainly employed in the private sector.^19,20^ NSSF usually receives 20% of an employee’s salary - equally shared between employee and employer. Members have to register for the healthcare benefit (SHIB), and no additional payments are made by the members. iCHF a voluntary scheme, targets the informal sector workers.^20^ The NHIF, SHIB, and iCHF operates under provider-purchaser spilt and members have access to public and private healthcare providers based on scheme arrangements.^19-21^ NHIF and SHIB funds are pooled at the national level while iCHF funds are pooled at the regional level where the regional administrative secretary (RAS) acts as a purchaser.^22,23^ NHIF and SHIB administrative functions are vested with the Board of Directors while for the iCHF they are vested with the respective RAS.^24^ Private health insurance firms are mainly concentrated in urban areas and target private-sector employees and wealthy individuals.^20^ Purchasing arrangements and functions for insurance schemes are guided by legislation that provides the requirements to establish, manage, and administer a scheme.^22,25,26^

## Methods

###  Study Setting

 We purposively selected three health insurance schemes NHIF, SHIB, and iCHF taking into consideration the variability in the targeted population, purchasing arrangements and functions, and potential to expand coverage to the population to achieve UHC (Table 1). We excluded tax-based financing as there is no purchaser-provider split,^17^ user fees because these resources are not pooled and their impoverishing effect,^27,28^ and programs like results-based financing which are not implemented country-wide and rely on donors. The study also considered data availability, ease of communication with the institution for clarification, and validation of information collected from various documents reviewed.

**Table 1:** Financial Management of the Three Schemes

**NHIF**	**SHIB**	**iCHF**
Formally employed contribute 6% of their monthly salaries, equally shared between the employee and employer for a household of 6 beneficiaries. Annual premium for under 18 years of age is TZS 50 400 (US $21.73) while for those above 18 years annual premium ranging TZS 192 000 (US $82.78) to 1 908 000 TZS (US $853.67) based on their benefits package and age.	Part of 20% of salary contributed per month, equally shared between the employee and employer as pension serving. Insurance benefit is for a household of 6 beneficiaries. Retired employee who wish to proceed with the insurance benefit are charged 6% of their pension.	Members contribute TZS 30 000 (US $12.93) for a household of 6 beneficiaries, except in Dar es Salaam where individual members pay TZS 40 000 (US $17.25).
Funds are pooled at the central level	Funds are pooled at the central level	Funds are pooled at the regional level
Covers 8% of the population	Covers less than 1% of the population	Covers about 6% of the population
Mandatory for public servants	Voluntary	Voluntary
Fee for service	Capitation adjusted based on the number of members registered and fee for service for referral care	Capitation adjusted for utilization (60%), enrolment (30%) and catchment population (10%)
Managed by the NHIF board	Managed by the NSSF board	Managed by the Office of RAS

Abbreviations: NHIF, National Health Insurance Fund; SHIB, Social Health Insurance Benefit; iCHF, improved Community Health Fund; NSSF, National Social Security Fund; RAS, Regional Administrative Secretary.

###  Study Design

 The study team used the multi-case qualitative study drawing on the three healthcare financing mechanisms (NHIF, SHIB, and iCHF) to explore the purchaser-provider relationship with a focus on purchasing arrangements and functions in Tanzania. The approach is considered appropriate in exploring a complex phenomenon in a real-life situation.^29^

###  Data Sources and Collection

 Data collection was guided by the strategic healthcare purchasing (SHP) progress mapping framework co-developed by the Strategic Purchasing Africa Resource Center (SPARC) and technical partners.^30^ Data were drawn from document review and validated with key informant (KI) interviews. In this SHP progress mapping, we zoomed in on the purchaser-provider relationship. We specifically focused on the following purchasing arrangements and functions: (1) the benefit specification (what to buy), (2) selective contracting (where to buy), (3) provider payment (how to buy), (4) monitoring provider performance (Table 2).

**Table 2:** Strategic Purchasing Functional Capacities

**Purchasing Functions **	**Purchasing Capacities **			
Benefits specification (Decide what to buy)	Specify benefits packages, including service delivery standards and cost-sharing policies	Communication	Development, operation, and effective use of IT system	Strategic planning and policy development
Contracting (Decide from whom to buy)	Select providers; enter into contracts to deliver goods and services in the benefits package			
Provider payment (Decide how and how much to pay providers)	Develop and implement provider payment systems and calculate payment rates			
Monitoring (Know how the money is being used)	Monitor provider performance, service utilization, and quality			

Abbreviation: IT, information technology.

####  Document Review

 Document review aimed to capture information on the country’s strategic purchasing arrangements and functions from the NHIF, SHIB, and iCHF. Documents were selected because of their content accuracy in relation to strategic purchasing functions, accessibility, and policy relevance. Documents included institutional annual reports, policy documents, and minutes from various health financing technical working grouping meetings (Supplementary file 1, Table S1). We also reviewed published documents related to purchasing arrangements and functions. It was important to complement and validate information reviewed with *KI interviews*.

####  Key Informant Interviews 

 Document review informed the purposive selection of the stakeholders for the KI. The interview guides were developed in English and translated into the local language. Interviews were conducted in English and in local language (Swahili) depending on the participants choice. Interviews were audio recorded with the permission of the study participants. Nine KI were conducted face to face with various stakeholders including government representatives and health insurance managers between August and September 2019 (Supplementary file 1, Table S2). Stakeholders were selected based on their experience in governance and financing of the schemes; laws, and regulations; contracting and payment arrangements; scheme monitoring; and overall service delivery.

###  Data Analysis 

 Data analysis was iterative and was guided by the SHP progress mapping framework. We started with the analysis of information from document review and then information from the interviews. Thematic analysis was used, using both deductive and inductive coding in the analysis of the documents and KI. Initial coding of the transcripts was carried out with two experienced researchers separately (both PhD candidates in health financing). Thereafter they exchanged the documents and discussed the findings together with the principal investigator. Any disagreement in the coding process was discussed with the senior person who was involved in the conceptualization of the study. Data were triangulated between the two sources (document review and KI). Documents were analyzed manually, while NVIvo version 12 was used for the KI analysis. The results have been organized according to the SHP arrangements and functions (see Table 2). The themes include (*i*) the benefit specification, (*ii*) selective contracting, (*iii*) provider payment and (*iv*) monitoring provider performance.

## Results

###  Benefits Specification 

####  The Variation in Services and Provider Access Across the Three Schemes

 The NHIF benefit selection was based on the standard treatment guidelines issued by the Ministry of Health. NHIF offers a broad range of services including, outpatient, in-patient, and specialized care as stipulated in the NHIF Act, Cap 395.^24,25,31^ NHIF members and beneficiaries are free to choose a service provider. SHIB members are entitled to outpatient and inpatient care at only one preferred provider with a referral if needed, however, admission services are limited to 42 days per year. SHIB benefits are stipulated in the NSSF Act of 1997.^26^ Similar to NHIF, SHIB uses standard treatment guidelines issued by the Ministry of Health. Healthcare services are limited to the medical services provided by doctors, nurses, and other medical providers in the accredited hospitals.^26^ SHIB beneficiaries are allowed to change healthcare providers in case one of change in residence. In such a scenario a beneficiary would fill in a special form and the scheme would shift the clients’ file to the preferred healthcare facility. This process is illustrated by the SHIB representative who said:

 “…*there is a registration form which a member has to fill information together with family information. Thereafter scheme beneficiaries will be required to select one healthcare provider based on member preference, in which s/he will be accessing services, we encourage them to select facilities close to their residence for easy access. The information is then processed and shared with the preferred healthcare provider. We inform the healthcare provider that the scheme member and dependants will be accessing care from the selected facility*…” [KI-3, Dar es Salaam].

 Likewise, to NHIF and SHIB, the iCHF benefit selection was based on the national essential health benefits package formulated by the Ministry of Health. iCHF beneficiaries are entitled to a basic package of curative and preventative healthcare services available at the primary healthcare facilities and referral for inpatient care. In most cases, beneficiaries’ access to healthcare services at the district and regional hospitals require a referral letter from primary healthcare facilities.^22^ There is co-payments for iCHF members when accessing healthcare services at the district and regional referral hospitals.

###  Selective Contracting 

####  Accreditation and Contracting Private for and Not for Profit Providers 

 NHIF has accredited all the public healthcare facilities in the country for the provision of services to NHIF beneficiaries by default. Also, some of the private for-profit and not-for-profit have signed the memorandum of understanding with the NHIF to provide services to the beneficiaries. More than 7700 health facilities have been accredited and contracted countrywide ensuring access to healthcare services for the members. Moreover, NHIF has extended service provision to its beneficiaries by signing the contract with private pharmacies and accredited drug dispensing outlets (ADDOs) to ensure access to healthcare commodities whenever there are stock-outs at the service delivery point. NHIF contracts are the basis for establishing service delivery standards, provider performance targets, and monitoring service provision. The contracts have improved access to care and incentivized providers to comply with the agreements, such as ensuring skilled staff are available and adequate to meet beneficiaries’ healthcare needs.

 The process of accrediting and contracting private providers could start from the provider or NHIF beneficiaries’ side. At times NHIF beneficiaries may request accreditation of a private healthcare facility based on their location and suggestions received during meetings with the stakeholders. Initiatives for the contractual agreement could start from the provider requesting to enter into a contractual agreement. While the private facilities upon showing interest in providing healthcare services to the NHIF beneficiaries undergo quality assessment. Thereafter, a memorandum of understanding is signed between the NHIF and the provider. The contract contains terms and conditions for services including price level for various services. The NHIF official explained this process:

 “*…initially when we started, NHIF role was not well known, we used to approach the healthcare providers, and inform them that we would like to work with them, but at the moment NHIF roles and function are known. So in case a person/institution has established a healthcare facility she just comes to us with the request, we do send a team of experts which goes and inspect the facility. Thereafter, NHIF board convene and discuss the request as well as the facility inspection report, once approved the facility is informed, and a formal contract is signed…*” [KI-1, Dar es Salaam].

 Contrary to NHIF, SHIB has contracted a limited number of public, and private healthcare providers across the country. In 2013, there were about 350 public and private healthcare providers who were contracted to render services to the SHIB beneficiaries country-wide.^32^ Private pharmacies and ADDOs are not contracted by the scheme. Moreover, scheme beneficiaries’ as well as providers have no room to approach the scheme and sign a memorandum of understanding. Selection and contracting of the providers are based on the strategic location of the provider, a range of services available as stipulated in the SHIB guideline, the willingness of the provider to enter into a service agreement with the SHIB as the purchaser.

####  Service Agreement With Faith-Based Organizations

 iCHF has accredited all the public primary healthcare facilities within the region by default and beneficiaries are eligible in accessing healthcare services from all public healthcare facilities.^33^ Furthermore, in some regions, iCHF beneficiaries have access to referral faith-based facilities through a contractual service agreement between the office of the RAS and owners of such non-governmental facilities.^33^ The contracts specify the types of services to be purchased from private not-for-profit healthcare facilities and the role of the office of RAS. Private not-for-profit facilities are contracted by the office of RAS because of the lack of public hospitals in the districts, and the willingness of the provider to enter into a contractual agreement based on the reimbursement negotiations. Furthermore, contracts with private not-for-profit facilities may arise from community preference and trust with the providers. When discussing the selection of the private providers for contractual agreements this was said:

 “*…the main thing which scheme beneficiaries wanted was private providers because in this region most providers are private, they are like 35 percent of all the healthcare providers. More important is not just the number of providers, but also the trust that people have with the providers. In the region majority of the people are religious people and they wanted to use the faith-based facilities. So we discussed with the office of the President’s Office Regional Administration and Local Government (PO-RALG) and we were allowed to enter into a contract with the providers…*” [KI-3, Dar es Salaam].

 It was further mentioned that the RAS may request for service agreement with the private facilities whenever the need arises. However, in all the contractual agreements the RAS agrees on behalf of the PO-RALG. This was highlighted during the discussion with the representative of the PO-RALG, who said:

 “*…few faith-based facilities are engaged within the iCHF initiatives. The faith-based facilities do enter into a contractual agreement with their respective regional administrative secretary as a representative/on behalf of the PO-RALG. The regional administrative secretary becomes the purchaser of the healthcare services…*” [KI-5, Dodoma].

###  Provider Payment

####  Fee-for-Service 

 Initially, the NHIF used capitation and fee-for-service to reimburse the healthcare providers for providing healthcare services to its beneficiaries, but at the moment has been using only fee-for-service. Providers are reimbursed after submitting monthly claims to the nearby NHIF regional office. Claim value below 200 million Tanzania Shillings is reimbursed by the regional office, while above 200 million are reimburse from headquarter. The provider has to specify all the service rendered to the scheme beneficiary and their respective fees charged within the claim form. NHIF staff take the responsibility of reviewing the documents before settling the claim. When discussing the provider payment mechanisms it was mentioned for instance:

 “….*Before 2012, we had a mix of payment methods (i.e. capitation and service fee). Claims were paid as one bundle, but there were some challenges in analyzing some of the services delivered to the members. Thereafter we had to switch/migrate from capitation to fee-for-service*…” [KI-2, Dar es Salaam].

 The NHIF has its price list for various services rendered to its beneficiaries and has been used by public and private providers. The prices vary by level of care and type of service provided. The prices/fee were established through the review of various policy documents/reports, actuarial valuation, costing study for assessing the cost of delivering health services, and technical advice.^31^ Determination of the fee rates was also informed by fund sustainability, the duration of service, and beneficiaries’ service utilization. It was reported that fee/prices charged per service could also be adjusted based on provider complaints. Prices charged for the healthcare commodities (drugs, medical equipment, and supplies) are based on the Medical Store Department price catalog. The price list is usually reviewed whenever the need arises and prices are amended accordingly. One of the KI participant when elaborating on the rates charged for various services within the NHIF package said:

 “…*we have a research team that visits the healthcare providers and collects information on the costs for various services offered by the respective provider. Then we compile the information and come up with the price/fee. Not only that but also once we have the prices/fees, we do share with various stakeholders (Ministry of Health, a private association of healthcare providers, healthcare providers) who review and give feedback before execution*…” [KI-1, Dar es Salaam].

####  Healthcare Equipment Loan 

 In addition to the fee-for-service reimbursed to the healthcare providers, NHIF has also entered into agreements with the providers where providers are eligible to request facility improvement and healthcare equipment loans. The aim is to improve service provision at the facility level as well as the surrounding environment. It was reported that NHIF has been lending the facilities loans for renovation or infrastructure improvement and its repayment is usually through deductions in facility monthly reimbursements. When explaining how loans are issued and recovered, the NHIF managers said:

 “…*we have the project named facility improvement and equipment loan, where we do lend to the facilities for renovation, either building a new building or renovation of a building to improve the environment, also purchase of the healthcare commodities which are needed at the facility to carter for the different need of the scheme beneficiaries, in turn, we make some deductions from the facility monthly reimbursements for a certain duration and some of the facilities have benefited through this process*…” [KI-1, Dar es Salaam].

####  Capitation 

 Contrary to NHF which used fee-for-service, SHIB has been using a capitation mechanism which is adjusted based on the number of members registered with a particular provider. The capitation amounts are being disbursed quarterly. Furthermore, SHIB uses a billing system per visit to reimburse the provider offering referral services to the members. When discussing the capitation and fee per visit as the two payment modalities used by the scheme, the scheme representative said:

 “…*capitation has been the basic payment to all the providers. The fee per visit was initiated after allowing scheme beneficiaries to access healthcare services from a higher level facility through the referral system. The healthcare provider cannot be reimbursed through capitation as the scheme beneficiary was not registered with that specific healthcare facility*…” [KI-3, Dar es Salaam].

 The representatives from the SHIB confirmed that capitation amounts are being determined based on the members’ utilization rates. It was reported that initially a survey was conducted to establish the base for the capitation amounts, and are revised every two years to adjust for inflation. When explaining how provider payment rates are conducted, the SHIB representative said:

 “…*capitation has a formula and has some calculations based on the utilization rates existing in a market. In the beginning, they conducted research and established the utilization rates, which helped to estimate the capitation amounts. After every two years, we are supposed to review the capitation rates/amounts, if there happens to be inflation, then the prices for drugs and service fee changes, this will lead to the change in the capitation amounts, if no inflation the capitation amount remain the same*…” [KI-3, Dar es Salaam].

 iCHF uses capitation to reimburse healthcare providers for the provision of healthcare services to the members.^34^ Capitation amount has been set per healthcare facilities based on the healthcare utilization by iCHF members, population enrolment rates into the scheme, and population catchment. For example, rates reimbursed to the primary healthcare providers takes into account 70% of the people utilizing healthcare services per facility, 20% of the number of iCHF beneficiaries enrolled with the scheme at a given healthcare facility, and 10% of the facility catchment population irrespective of being iCHF member.^22^ Contracted private not-for-profit healthcare facilities are paid on a capitation basis based on the agreement with the office of the regional and administrative secretary.

###  Provider Monitoring 

####  Monitoring Provider Through Quality Assurance of Clinical Health Services

 NHIF uses two ways to monitor the performance of the healthcare providers as outlined in the NHIF Act, through quality assurance of clinical health services and monitoring service provision. NHIF Act sect 26 outlines clinical quality monitoring where providers have to make sure that the quality of healthcare services is delivered following the standard treatment guideline provided by the Ministry of Health. While NHIF Act sub 27 (l) performance monitoring of service provision is done through a contract. There are periodic assessments, and these assessments also guide the decision to add a benefits package or remove some services. In each region, NHIF has a quality assurance manager whose responsibility is to conduct regular inspections of the facilities to ensure providers deliver quality services to the NHIF beneficiaries. When explaining how inspection and verification are conducted, the NHIF managers said:

 “…*monitoring is done, for example, there are routine inspections conducted after reviewing the provider’s claim forms. We do verify whether the provider rendered services that match with the claim amounts, or is it true that the scheme beneficiaries have received the services as specified within the claim form, whether the providers keep records of the services rendered to scheme beneficiaries, using normal paper files or in the electronic system*….” [KI-2, Dar es Salaam].

 NHIF monitoring system aims to safeguard against unnecessary diagnostic and therapeutic procedures and intervention; over-utilization or under-utilization of healthcare services; irrational medication and prescription; and inappropriate referral practices.^25^ During the visit at the facility level, the quality assurance manager does meet with the beneficiaries accessing services and discuss with them the quality of services they receive from the facilities. Among the tools that they have been using are the standard national guidelines for service provision.

 Initially, NHIF started with paperwork for most of the services provided, and later on migrated to an electronic system for registration, verification of the beneficiaries, and claims. Most of the facilities have been using the electronic system, with some that are still using paper-based for claiming. When explaining how NHIF beneficiary information on contribution and healthcare utilization is being captured, the NHIF managers said:

 “…*we have various information system which is in place. We have an information system for keeping beneficiary information on contribution together with their dependents. Also, we have a claim system, which is being used to document all the claims from healthcare facilities, showing services that have been used by beneficiaries and the amount claimed for services. There is another information system used for verification at the point of service to retrieve information on the activeness of the member in the scheme. There is another system for issuing permits for service provision as there are categories of members who do not qualify for certain services based on enrolment category*….” [KI-1, Dar es Salaam].

####  Monitoring Provider Through Annual Assessment 

 In addition to the regular inspection of the facilities, the NHIF conducts an annual assessment of healthcare providers after every three years to assess whether healthcare facilities abide by the contractual agreements. Healthcare providers are given feedback on the assessment and suggestions on ways to improve service provision are put forward. In the case of misconduct such as fraud, NHIF may issue a warning letter to the provider or cancel the contract with the providers. District medical officer and district executive director are responsible to oversee that the public facilities adhere to the rules, regulations, and suggestions put forward after annual assessment are taken up.

 At times NHIF staff do visit providers to validate claims and services provided through checking into facility registers. In some instances, NHIF staff call the beneficiaries who received services to validate the service provided to them. Moreover, NHIF has medical specialists who review the service rendered to the beneficiaries in the claim forms and compared it with the stated illness conditions described. If the claim document has some missing information or anything that is not clear, has to be returned to the provider for amendment. In a few cases, NHIF does not reimburse the provider the full amount claimed if there a lot of doubts (such as a fraud case). During KI it was reported that staff does examine whether claim documents submitted to the NHIF and whether the prices charged per services are in line with the contractual agreement. It was further reported that in case a facility has made false claims a contract may be terminated for the reputation of the purchaser.

####  Monitoring Provider Through Supportive Supervision 

 Whereas, SHIB has been assessing provider performance through supportive supervision done at least twice a year. They do visit the healthcare providers with a special checklist to assess the quality of service provision. We found that such visits help in deciding on contract maintenance with the provider and at times interviews are conducted with clients to get complaints about service provision. Information is used to advise the providers to consider upgrading their service provision and in this way, they can also benefit from an adjustment in capitation amount. Moreover, after every two years, SHIB reviews utilization rates of the facilities to be able to determine the rate of capitation to be disbursed to providers. If there are no changes in the utilization rates, the capitation amount remains the same. If there are changes in utilization and inflation rates on medicine prices and service fees, adjustment is made on the capitation. When explaining how information is being processed and shared with the prodder, the SHIB representative said:

 “…*The information is processed using the computer system and the reports are being generated and shared with the preferred healthcare providers. When accessing healthcare services, the provider uses the shared file to verify whether the scheme member is eligible in accessing healthcare services based on the online database*…” [KI-3, Dar es Salaam].

 Monitoring provider performance under the iCHF is done through Regional Health Management teams and Council Health Management Teams. The teams conduct supportive supervision visits to all public facilities together with the contracted healthcare facilities. Moreover, the Ministry of Health through its quality assurance department does visit the healthcare facilities to assess the quality of services provided by the healthcare facilities based on the service provision standards specified by the Ministry of Health.

 The healthcare facility in-charges submit monthly and quarterly reports on iCHF members’ healthcare utilization to the district headquarters. The information is summarized and recorded into the district health management information system and used to monitor trends in healthcare utilization for the iCHF members.^35^ Also, the system provides information that is being used for adjustments of the healthcare provider payment formula. iCHF contracted healthcare facilities for referral services submit monthly reports to the RAS including the value of the healthcare services delivered to all iCHF patients. Capitation funds disbursed to the contracted healthcare facilities have been monitored through the monthly reports and subsequent payment has to be done after the respective healthcare facility use 90% of the funds.

## Discussion

 This study analyzed the purchaser-provider relationship, focusing on purchasing arrangements and functions for three health financing schemes in Tanzania. The strategic health purchasing progress mapping has shown that all the schemes have been implementing a purchaser-provider split.

###  Financial Sustainability

 NHIF offers a broad benefits package to its members and has been successful in expanding benefits packages because of the strong organization arrangements, revenue-raising, pooling, and mechanisms in place used to pay for the services. The success has been attributed to the fact that NHIF enrolment is compulsory for public servants and contributions are progressive.^18^ Assurance in the revenue generation and pooling helps in strengthening the strategic purchasing functions of the scheme. Even though SHIB contributions are proportional to members’ income and pooled at the national level, the benefits package is limited because offering health insurance is not the core business of the scheme. Moreover, there are no earmarked contributions specifically for the reimbursement of healthcare providers, SHIB is financed through the NSSF contributions.^36^ Strategic purchasing is also limited by the fact that not all the members of the NSSF are enrolled in the SHIB, enrolment is based on the individual willingness to access healthcare services through SHIB. Assessment of the private sector conducted in 2013 showed that only 10% of the NSSF members had registered for the SHIB benefit.^32^ iCHF benefit is limited because of the voluntary nature of the scheme and this makes it difficult in projecting revenue generation in a given fiscal year.

###  A Broader Choice of Provider

 NHIF has accredited many facilities. However, there are some elements of inequity as most of the accredited providers which offer specialized services are found in urban settings where members have to travel a distance to access such services. Also, there is the existence of quality differences between accredited healthcare providers for primary care facilities.^37^ It is important to note that NHIF accreditation of the private pharmacies and ADDOs aimed at addressing the challenge of drug shortages, especially to the public healthcare facilities whenever NHIF members access services. To access drugs at the pharmacies NHIF members have to be referred from the accredited healthcare provider. Accreditation of drug dispensing outlets has been successful in improving the availability of drugs, nonetheless, there are still some challenges related to administration and claims management.^37^ SHIB has a limited number of contracted healthcare providers which are not evenly distributed countrywide.^32^ This may pause a challenge for beneficiaries in accessing healthcare services. Compared to NHIF and SHIB, the office of the RAS has not managed to contract private for-profit for the provision of healthcare services for iCHF beneficiaries because service charges for the private providers are still higher to the extent that the scheme may not be able to reimburse the costs of the services.

###  Provider Payment

 The three financing mechanism uses a different system for the payment of the provider, the fee-for-services for NHIF, capitation for SHIB and iCHF. iCHF capitation formula has some adjusters including enrolment rates, utilization, and population catchment.^22^ One should take note that each of the provider payment methods chosen has some pros and cons. They do affect service provision and at times may create incentives to achieve policy objectives including equitable access to quality health services and efficiency in healthcare service provision towards achieving UHC.^38^ For example, fee-for-service has been found to improve access to services, but in certain cases may also increase the risk of overtreatment^39,40^; while capitation does promote healthcare provider efficiency, low administrative costs. SHIB has contracted few healthcare providers, this may create a crowding effect, affect the quality of care, and creates inequity as providers may tend to attract more clients to attract more revenue through capitation.^41^ Fee-for-service, as used by NHIF if not closely monitored, may lead to inefficiency in service delivery as may open up for over-servicing and increased access to certain services. Nonetheless, a lack of efficient control mechanisms for some providers may choose healthier patients or undertreat patients which jeopardizes access to quality healthcare services for the marginalized population.^42^ It is important to set some strategies to mitigate such cons associated with such reimbursement mechanisms including legal, regulatory, quality controls, effective gatekeeping process, and deployment of technology for effective mitigation of risks/fraud.

###  High-Cost Curative Services at the Expense of Primary Care

 A recent analysis of provider payments revealed that referral hospitals receive more than two-thirds of NHIF reimbursement.^37^ Furthermore, it was found that most of the NHIF beneficiaries seek care from public providers, however, only about one-third of the NHIF reimbursement goes to the public providers. NHIF beneficiaries utilize services from private providers and this might be due to the utilization of complex procedures and specialized care. This also could reflect the fact that public providers have no incentives to improve healthcare services. Payment of providers is according to delivery of services and not inputs this method does incentivize providers to improve utilization and efficiency. The move towards a system that finances outputs is important for the country towards strategic purchasing arrangement and functions.^37^ NHIF has managed to improve efficiency in service delivery through the provision of medical equipment and facility improvement loans. This offers the opportunity for improvement in the quality of service offered to the members and providers may leverage the use of the resources to provide services to the public in general. Providers may make use of this strategic option as a means of ensuring the provision of services that are needed by the beneficiaries. NHIF gatekeeping of access healthcare services by controlling card use and authorization promotes rational use of the healthcare services for the higher level of care.

 In Tanzania, several policy documents have been prepared to guide the country towards UHC. Among them includes the Health Sector Strategic Plan for 2015-2020^11^; the health financing strategy 2016-2026 ^23^ and healthcare policy, 2017.^43,44^ The health financing has shown some of the healthcare reforms taking place in the country including the scale-up of the coverage of redesigned community health funds – iCHF.^45^ The health sector strategic plan emphasizes the importance of creating innovative strategies for establishing, maintaining, and sustaining public-private partnerships in service delivery. While the health financing strategy highlights the government goal of establishing a mandatory Single National Health Insurance (SNHI) under which the entire population of Tanzania will have access to a standard minimum healthcare benefits package at all levels of care.^23^ It is anticipated that the expansion of social healthcare protection will improve household access to care and facility revenue which providers could use to improve the availability of healthcare commodities and improve in facility environment.^46,47^ Other reforms include the implementation of direct health facility financing^48,49^ and facility financial accounting and reporting system.^49^ Initially, most of the health centers and dispensaries had no bank accounts. All the cost-sharing funds (user fees, basket funds, and insurance reimbursements) were managed and controlled at the district level, and facilities had no direct access to funds or control of financial resources.^49^ In 2011/2012, all public primary facilities were directed to open a bank account with a local/nearby bank that was approved by the Bank of Tanzania and cost-sharing funds are deposited in the accounts. In 2017/2018, the government allowed for fiscal decentralization, and health basket funds are transferred directly to each healthcare facility bank account.^49^ The re-design of the community health insurance would improve access to health for the households as well as revenue to the healthcare facilities. These reforms are expected to increase health facility autonomy, improve purchasing arrangements for essential medicines, and facilitate operational costs at the facility level.^48,49^ Implementation of DHFF is expected to improve accountability and governance, increasing health system responsiveness and improving health-seeking behaviour and service utilisation at the primary health facility level.^49^ Financial accounting and reporting system have been used to track and monitor funds flowing to public healthcare providers as well as expenditure on various items including the purchase of healthcare commodities at the healthcare level.

 In this study, there was no much information on the power of the healthcare providers over purchasers which in one way or the other does influence SHP. The provider power could be exercised through lobbying strategies and the ability to negotiate service delivery to the clients with the purchasers. Most of the providers do provide healthcare services to other groups of the people in the community, and at times they do compare the revenue generated from other sources such as user-fee which in one way or the other may incentivize them more compared to the contractual agreements with the purchasers.

 The findings for this case study should be interpreted with the following limitations. The qualitative approach used in document review and KI limits the statistical generalization of the findings within and outside Tanzania. Only two scientists participated in document review and analysis, this has a potential for misinterpretation of the findings. The field would benefit from having beneficiaries/citizen feedback on access and utilization of healthcare services as well as their involvement in the identification of their healthcare needs for inclusion in the benefit packages including the choice of the provider. A future study could consider the involvement of the beneficiaries/citizen in getting opinions and experience of the healthcare service delivery would have enriched the findings of this study and strengthened policy recommendations.

## Conclusion

 NHIF is the largest purchaser of healthcare services, the purchasing functions should be prioritized. The NHIF being mandatory for public servants and resources being pooled centrally creates assurance in the revenue generation and helps in strengthening strategic purchasing functions of the scheme. NHIF offers a broad benefits package to its members hence improving access to services countrywide, however, accreditation of the specialized care should be reviewed to address geographical inequity. Also, NHIF needs to monitor quality differences between accredited healthcare and claims management, especially for the primary healthcare facilities. The government intention towards SNHI not only calls for harmonization of the health insurance schemes across the country but also the healthcare purchasing functions including resources collection and pooling; healthcare benefits provided; payment mechanisms and data management systems. This will help to improve efficiency in the allocation and use of funds. Furthermore, investment in a routine monitoring system, such as the use of the district health information system which allows effective tracking of healthcare service delivery, and broader population healthcare outcomes. Communication of healthcare strategic purchasing arrangements and functions is crucial for the understanding of the interactions and accountability of the purchasing functions to relevant stakeholders, including the government, insurance schemes, and healthcare providers as well as the citizens. Enforcing compliance with the contractual agreement between providers and purchasers is crucial for the provision of quality services in an efficient manner. Strengthen coordination between the entities managing the purchasing functions is important in improving access and utilization of quality healthcare as well as in ensuring equity. As some of the healthcare financing reforms are taking place in the country, purchasing functions should be reviewed to increase the potential in accelerating the country’s progress towards UHC.

## Acknowledgments

 We would like to acknowledge the assistance of the institutions that participated in the KI interviews. Special thanks to Agnes Gatome-Munyua, for her critical review of the paper. We also acknowledge the SPARC together with Results for Development (R4D) for technical support and for funding the study.

## Ethical issues

 Ethics approval was obtained from the Ifakara Health Institute review board (IHI/ IRB/No. 45-2020) and National Institute for Medical Research (NIMR) (NIMR/HQ/R.8a/Vol IX/3909). All study participants for the KI were informed, and verbal consent was obtained from the participants. Confidentiality of the participant’s information was assured and data anonymity was considered during data collection and analysis.

## Competing interests

 Authors declare that they have no competing interests.

## Authors’ contributions

 SM and FM conceptualized the study. AK participated in data analysis, interpretation of data and drafted the manuscript; SM and PB substantially contributed to data acquisition; SM and FM participated in interpretation of results. All authors have critically reviewed the manuscript and approved the final version.

## Funding

 This work was funded by the Bill and Melinda Gates Foundation through the SPARC - a resource hub hosted by AMREF Health Africa with technical support from Results for Development. SPARC aims to strengthen strategic purchasing capacity in Sub-Saharan Africa by connecting existing regional expertise and matching it with country demand to make better use of health resources. SPARC co-created, with a consortium of Africa-based Anglophone and Francophone partners, a common SHP progress mapping framework. The partners populated an excel tool based on the SHP progress mapping framework to map strategic purchasing governance arrangements, functions, capacities, and results. The consortium of partners includes Ifakara Health Institute, University of Dar es Salaam, Makerere University School of Public Health, University of Rwanda School of Public Health, KEMRI Wellcome Trust Programme, Centre de Recherche en Reproduction Humaine et en Demographie (CERRHUD– Benin), Kwame Nkrumah University of Science and Technology (KNUST– Ghana), Health Policy Research Group (HPRG – Nigeria), Formation, Recherche, Etude et Conseil en Couverture Sanitaire Universelle (FRECSU – Burkina Faso), UCT Health Economics Unit (South Africa) and Research for Development International (R4D International – Cameroon). This manuscript is an output from Ifakara Health Institute which participated in the mapping of Tanzania’s purchasing arrangements.

## Supplementary files

Supplementary file 1 contains Tables S1-S2.
