# Supplementary material for: Assessment of Strategic Healthcare Purchasing Arrangements and Functions Towards Universal Coverage in Tanzania
Source: Int J Health Policy Manag. 2022 Aug 1;11(12):3079–89. doi: 10.34172/ijhpm.2022.6234 (PMC10105173; doi:10.34172/ijhpm.2022.6234)
Supplement: Supplementary file 1 — contains Tables S1-S2. [file ijhpm-11-3079-s001.pdf]

Article title: Assessment of Strategic Healthcare Purchasing Arrangements and Functions Towards Universal Coverage in Tanzania

Journal name: International Journal of Health Policy and Management (IJHPM)

Authors' information: August Kuwawenaruwa*, Suzan Makawia, Peter Binyaruka, Fatuma Manzi

Ifakara Health Institute, Dar es Salaam, Tanzania.

(corresponding author: ajoachim@ihi.or.tz)

Supplementary file 1

Table S1: Documents reviewed in the study

Number	Name of the document	Year
1	The National Health Policy 2017	2017
2	Health Sector Strategic Plan IV (HSSP IV) July 2015 – June 2020, Reaching all Households with Quality Health Care	2015
3	Community Health Fund Act, 2001.	2001
4	The National Health Insurance Fund Act, 1999.	1999
5	The National Social Security Fund Act, 1997	1997
6	Tanzania Health Financing Strategy 2016-2026 Path towards Universal Health Coverage, Final Draft	2016
7	Direct Health Facility Financing (DHFF) & Roadmap Presented On 06.06.2017	2017
8	National Health Accounts For Financial Years 2013/14, 2014/15 And 2015/16. The United Republic Of Tanzania Ministry of Health, Community Development, Gender, Elderly And Children	2019
9	Health Sector Public Expenditure Review 2016/17	2018
10	Direct Health Facility Financing as a practical approach for strengthening strategic purchasing in Tanzania	2019
11	Tanzania Health Insurance Regulatory Framework Review	2012
12	Health Sector Strategic Plan IV, Mid Term Review Presentation of Finding, Dar es Salaam – Tanzania	2019
13	Options for Expanding Private Sector Contributions to Health. Bethesda, MD: Strengthening Health Financing Capacity, Tanzania Project	2013
14	Options For Health Insurance Market Structuring, For The Tanzania Health Financing Strategy, Final Report	2013
15	Health Care Financing Strategy Background Paper #1: Options for the Minimum Benefits Package in Tanzania.	2013
16	Tanzania Health Insurance Institutional Assessment In support of Health Financing Strategy Development, Dar es Salam, Tanzania.	2013
17	Development of the Tanzania Health Financing Strategy; Options paper nr. 6. Reform options for the CHF systems, Final Report, Dar es Salaam, Tanzania.	2013
	Meeting documents	
18	Consultative Forum on Health Financing Strategy and National Health Insurance in Tanzania at Hotel Morena in Dodoma, Tanzania	2018
19	Meeting minutes from Tanzania Health Financing Technical Working Grouping Meeting (various)<br>i) Update on Implementation of CHF Iliyoboreshwa (iCHF)<br>ii) Minutes for health financing technical working group meeting held at CCM Conventional Hall – Dodoma, Tanzania<br>iii) Minutes for health financing technical working group meeting held at CCM conventional hall – Dodoma, Tanzania<br>iv) Ministry of Health, Community Development, Gender, Elderly And Children Health Financing TWG Meeting Dodoma, Tanzania	2020<br>2019<br>2019<br>2019

Table S2: Key informant interview participants

Institution	Department	Region	No. interviewed
NHIF	Regional Office	Dar es salaam	1
NHIF	Headquarter	Dodoma	1
NSSF	SHIB department	Dar es salaam	1
Pharm access	Manager	Dar Es salaam	1
PO-RALG	Representative	Dodoma	1
External Partners	HPSS Project	Dodoma	1
iCHF	iCHF department at NHIF office	Dar es salaam	1
Ministry of Health	Policy Department	Dodoma	1
Private Insurance	AAR	Dar Es salaam	1
